# Extracorporeal shockwave therapy in musculoskeletal disorders

**DOI:** 10.1186/1749-799X-7-11

**Published:** 2012-03-20

**Authors:** Ching-Jen Wang

**Affiliations:** 1 Department of Orthopedic Surgery, Section of Sports Medicine, Chang Gung University College of Medicine, Kaohsiung Chang Gung Memorial Hospital, 123 Ta-Pei Road. Niao Sung District, Kaohsiung City 833, Taiwan

**Keywords:** Extracorporeal shockwave therapy, Musculoskeletal disorders

## Abstract

The sources of shockwave generation include electrohydraulic, electromagnetic and piezoelectric principles. Electrohydraulic shockwaves are high-energy acoustic waves generated under water explosion with high voltage electrode. Shockwave in urology (lithotripsy) is primarily used to disintegrate urolithiasis, whereas shockwave in orthopedics (orthotripsy) is not used to disintegrate tissues, rather to induce tissue repair and regeneration. The application of extracorporeal shockwave therapy (ESWT) in musculoskeletal disorders has been around for more than a decade and is primarily used in the treatment of sports related over-use tendinopathies such as proximal plantar fasciitis of the heel, lateral epicondylitis of the elbow, calcific or non-calcific tendonitis of the shoulder and patellar tendinopathy etc. The success rate ranged from 65% to 91%, and the complications were low and negligible. ESWT is also utilized in the treatment of non-union of long bone fracture, avascular necrosis of femoral head, chronic diabetic and non-diabetic ulcers and ischemic heart disease. The vast majority of the published papers showed positive and beneficial effects. FDA (USA) first approved ESWT for the treatment of proximal plantar fasciitis in 2000 and lateral epicondylitis in 2002. ESWT is a novel non-invasive therapeutic modality without surgery or surgical risks, and the clinical application of ESWT steadily increases over the years. This article reviews the current status of ESWT in musculoskeletal disorders.

## 

Extracorporeal shockwave therapy (ESWT) began with an incidental observation of osteoblastic response pattern during animal studies in the mid-1980 that generated an interest in the application of ESWT to musculoskeletal disorders. In the past 10 to 15 years, shockwave therapy had emerged as the leading choice in the treatment of many orthopedic disorders including proximal plantar fasciitis of the heel [1-6], lateral epicondylitis of the elbow [7-10], calcific tendinitis of the shoulder [11,12] and. non-union of long bone fracture [13-15]. More recently, the use of ESWT had expanded to the treatment of patellar tendinopathy (jumper's knee) and Achilles tendinopathy [16-19], and avascular necrosis of the femoral head [20-22]. ESWT has gained significant acceptance from Europe (Germany, Austria, Italy and others) to South America (Brazil, Columbia, Argentina and others), Asia (Korea, Malaysia, Taiwan and others) and North America (Canada and USA), and this had led to the change of European Society for Musculoskeletal Shockwave Therapy to International Society for Musculoskeletal Shockwave Therapy (ISMST) in 2000. In USA, FDA (Food and Drug Administration) first approved the specific shockwave device, OssaTron (High Medical Technology, Lengwil, Switzerland, now Sanuwave/Alpharetta, GA) for the treatment of proximal plantar fasciitis in 2000 and lateral epicondylitis of the elbow in 2003. FDA also approved Epos (Dornier Medical System, Kennesaw, GA) for the treatment of plantar fasciitis and Sonocur (Siemens Medical Systems, Iselin, NJ) for the treatment of lateral epicondylitis of the elbow in 2002, Orthospec (Medispec, Germantown, MD) and Orbasone (Orthometrix, White Plains, NY) for the treatment of plantar fasciitis in 2005. In the meantime, many off-label uses of ESWT were also studied including calcific tendinitis of the shoulder, patellar tendinopathy, Achilles tendinopathy, and non-union of long bone fracture, avascular necrosis of the femoral head and others. The vast majority of the published papers including randomized control trials and cohort studies showed positive effects and evidence base medicine in favor of ESWT [1-6,8-12,23]. However, a few studies reported that ESWT is ineffective or less effective with the results comparable to the placebo effect [7,24,25], and this has stirred up the debate and controversy. This article reviews the current status of ESWT in the treatment of musculoskeletal disorders.

## Principle of shockwave generation

There are three main techniques through which shockwaves are generated. These are electrohydraulic, electromagnetic, and piezoelectric principles, and each of which represents a different technique of generating shockwaves. Electrohydraulic principle represents the first generation of orthopedic shockwave machine. Electrohydraulic shockwaves are high-energy acoustic waves generated by the underwater explosion with high-voltage electrode spark discharge, and the acoustic waves are then focused with an elliptical reflector and targeted at the diseased area to produce therapeutic effect [26]. It is characterized by large axial diameters of the focal volume and high total energy within that volume [27]. Shockwave generation through the electromagnetic technique involves the electric current passing through a coil to produce a strong magnetic field. A lens is used to focus the waves, with the focal therapeutic point being defined by the length of the focus lens. The amplitude of the focused waves increases by non-linearity when the acoustic wave propagates toward the focal point [26,27] Shockwave of piezoelectric technique involves a large number (usually > 1,000) of piezocrystals mounted in a sphere and receives a rapid electrical discharge that induces a pressure pulse in the surrounding water steepening to a shockwave. The arrangements of the crystals cause self-focusing of the waves toward the target center, and lead to an extremely precise focusing and high-energy within a defined focal volume. When comparing different shockwave devices, the important parameters include pressure distribution, energy density and the total energy at the second focal point in addition to the principle of shockwave generation of each device.

Shockwave pattern differs from ultrasound wave that is typically biphasic and has a peak pressure of 0.5 bar. Shockwave pattern is uni-phasic with the peak pressure as high as 500 bars [26]. In essence, the peak pressure of shockwave is approximately 1,000 times that of ultrasound wave. There are two basic effects of shockwave. The primary effect is the direct mechanical forces that result in the maximal beneficial pulse energy concentrated at the target point where treatment is provided; and the secondary effect is the indirect mechanical forces by cavitation which may cause negative effect or damage to the tissues [26-30].

## Mechanism of shockwave therapy

The mechanism of shockwave therapy is not fully understood. The most important physical parameters of shockwave therapy for the treatment of orthopedic disorders include the pressure distribution, energy flux density and the total acoustic energy. In contrast to lithotripsy in which shockwaves disintegrate renal stones, orthopedic shockwaves are not being used to disintegrate tissue, but rather to microscopically cause interstitial and extracellular responses leading to tissue regeneration [26,27].

## Animal experiments

### Shockwave therapy for bone healing

Several studies had investigated the effects of shockwave therapy on fracture healing and articular cartilage in animal experiments. Haupt et al in an experimental model in rats, confirmed a positive effect of shockwave treatment on fracture healing [31]. Johannes et al showed the promotion of bony union with shockwave therapy in hypertrophic non-unions in dogs [32]. Wang et al demonstrated that shock wave therapy enhanced callus formation and induced cortical bone formation in acute fractures in dogs and the effect of shockwave therapy appeared to be time-dependent [33]. Forriol et al, however, reached an alternative conclusion and thought that shockwave treatment might delay bone healing [34]. The conflicting results are due different types of animals and different shockwave dosages used. Wang et al had demonstrated that high-energy shockwave therapy produces a significantly higher bone mass including BMD (bone mineral density), callus size, ash and calcium contents, and better bone strength than the control group after fractures of the femurs in rabbits. The effects of low-energy shockwave therapy were less prevailing with comparable results as compared to the control. Therefore, the effect of shockwave therapy on bone mass and bone strength appeared to be dose- and time-dependent [35]. Many other studies also investigated the effect of shockwave therapy on bone healing in animals. The important findings included superoxide mediates shockwave induction of ERK-dependent osteogenic transcription factor (CBFA-1) and mesenchymal cells differentiation toward osteoprogenitors [36]. Extracorporeal shockwave promotes bone marrow stromal cell growth and differentiation toward osteo-progenitors associated with TGF-β1 and VEGF induction [37]. Physical shockwave mediates membrane hyperpolarization and Ras activation for osteogenesis in human bone marrow stromal cells [38], Shockwave promotes bone regeneration by the recruitment of mesenchymal stem cells and expressions of TGF-β1 and VEGF [39].

### Shockwave therapy for insertional tendinopathy

Many studies investigated the effect of shockwave therapy on insertional tendinopathies. Rompe et al demonstrated dose-related effects of shockwave on rabbit tendo Achilles, and suggested that energy flux density of more than 0.28 mJ/mm^2 ^should not be used clinically in the treatment of tendon disorders [40]. In their study, a statistically significant increase of capillary formation was noted with higher energy shock wave (0.60 mJ/mm^2^), which also caused more tissue reaction and potential damage to the tendon tissue. Wang et al had demonstrated that shockwaves enhance neovascularization with formation of new capillary and muscularized vessels at the tendon-bone junction of the Achilles tendons in dogs [41]. In another study in rabbit model, Wang et al further demonstrated that shockwave therapy induces the ingrowth of neo-vessels (neovascularization) including capillary and muscularized vessels than the control at the tendon-bone junction. Shockwave therapy releases angiogenetic growth and proliferating factors including *e*NOS, VEGF, and PCNA [42]. The *e*NOS and VEGF began to rise in as early as one week and remained high for 8 weeks, then declined to baseline in 12 weeks; whereas the increase of PCNA and neo-vessels began in 1 weeks and persisted for 12 weeks and longer. Therefore, the mechanism of shockwave therapy may have involved the improvement in agniogenetic growth factors, which in turn induce neovascularization and improve blood supply at the tendon-bone junction of the Achilles tendon in rabbits.

Chronic tendinopathy is an overuse syndrome manifested with pain and tenderness due to mucoid and chondroid degeneration and formation of plump tenocytes and increased fibroblastic and myofibroblastic cells and absent inflammatory cells [43]. Some studies reported that chronic painful tendinopathy exhibited increased occurrence of sprouting nonvascular sensory, substance P-positive nerve fibers and decreased occurrence of vascular sympathetic nerve fibers, and suggested that the altered sensory-sympathetic innervation may play a role in the pathogenesis of tendinopathy [44]. It is believed that shockwave therapy alleviates pain due to insertional tendinopathy by the induction of neovascularization and improvement of blood supply to the tissue, and initiating repairs of the chronically inflamed tissues by tissue regeneration.

### Clinical applications

#### Proximal plantar fasciitis

Many studies investigated the effect of shockwave therapy in the treatment of proximal plantar fasciitis and reported a success rate ranging from 34% to 88% [1,45-62]. The majority of the published papers reported a positive and beneficial effect of ESWT in proximal plantar fasciitis. Rompe et al suggested that three weekly treatments with 1,000 impulses of low-energy shockwave at 0.06 mJ/mm^2 ^appear to be an effective therapy for plantar fasciitis with significant alleviation of pain and improvement in function [58]. Wang et al treated 79 patients (85 heels) with plantar fasciitis including 59 women and 20 men with an average age of 47 years (range 15-75 years) with shockwave therapy. At one-year follow-up, the overall results were 75.3% complaint free, 18.8% significantly better, 5.9% slightly better and none unchanged or worse. The recurrent rate was 5% [60]. It was concluded that shockwave therapy is a safe and effective modality in the treatment of proximal plantar fasciitis.

In contrast, few studies reported the opposite results of ESWT in the treatment of plantar fasciitis [7,24,25,63,64]. Buchbinder R et al compared 81 patients who received ultrasound-guided ESWT given weekly for 3 weeks to a total dose of at least 1,000 mJ/mm^2 ^with 85 patients in the placebo group who received treatment to a total dose of 6.0 mJ/mm^2^, and concluded that no evidence to support a beneficial effect of ESWT over placebo on pain, function and quality of life [24]. Haake M et al compared 135 patients allocated to ESWT with 137 patients allocated to placebo and the results showed that ESWT is ineffective in the treatment of chronic plantar fasciitis [64]. In a randomised double blind control trial, Speed CA et al concluded that no treatment effect of moderate dose of ESWT in subjects with plantar fasciitis. Efficacy may be highly dependent upon machine types and treatment protocol [25]. Therefore, controversy exists on the effect of ESWT in the treatment of chronic plantar fasciitis. The differences are probably due to the difference in methodology of the study, the patient selection criteria, the use of different devices, different energy levels and the total energy and the outcome measurements.

Several studies compared the effect of ESWT with surgery, local corticosteroid injection or physical therapy in the treatment of proximal plantar fasciitis [62,65,66]. Surgical treatment by plantar fasciotomy and ESWT showed comparable functional outcomes, however, ESWT incurred no surgical risks including surgical pain [62]. Physical therapy has shown to be comparable or better effect than ESWT in proximal plantar fasciitis, however, physical therapy is time consuming and inconvenient [63]. Corticosteroid injection shows better short-term effect, but the long-term results favor ESWT [66].

The application of ESWT in proximal plantar fasciitis is performed with either local anesthesia or no anesthesia. Several reports showed that ESWT is less effective when the treatment is performed with the use of local anesthesia [67,68]. The majority of our patients were treated with no local anesthesia. However, our observations failed to distinguish any difference between treatment with or without local anesthesia. In case patient is unable to tolerate the procedure because of pain during treatment, the anesthesia with constant sedation can be used.

The complications of ESWT in proximal plantar fasciitis are low and negligible. Local reddening, ecchymosis, or mild hematoma, and migraine are among the list of complications. The complications can be successfully managed conservatively and spontaneous recovery is anticipated.

In summary, the literature review unveiled discrepancy and controversy on the effect of ESWT on proximal plantar fasciitis. Many factors can influence the effects of ESWT in the treatment of proximal plantar fasciitis. The vast majority of the published papers are in favor of ESWT. Additional studies are needed to validate the effectiveness of ESWT in the treatment of proximal plantar fasciitis.

#### Lateral epicondylitis of the elbow

Several studies investigated the effect of shockwave therapy in patients with lateral epicondylitis of the elbow, and the success rate ranged from 68% to 91% [69-75]. Rompe et al reported good or excellent outcome in 48% and an acceptable results in 42% at the final review at 24 weeks in 50 patients with chronic tennis elbow treated with 3,000 impulses of shockwave therapy compared with 6% and 24%, respectively, in the control patients treated with 30 impulses [76]. Wang et al compared the results of shockwave therapy in 57 patients (58 elbows) with lateral epicondylitis of the elbow with a control group of 6 patients (6 elbows) with a follow-up of 12 to 26 months. The overall results of the treatment group were complaints free in 27 (61.4)%, significantly better in 13 (29.5)%, slightly better in 3 (6.8%) and unchanged in 1 (2.3%). Recurrent pain of lesser intensity was noted in 3 patients (6.8%). In the control group, however, the results were unchanged in all 6 patients [77]. Few studies reported no effect of ESWT or less effect comparable to the placebo [78-83]. In a review of 9 placebo-controlled trials, Buchbinder et al concluded that there is "platinum" level that ESWT provides little or no benefit in term of pain and function in lateral elbow pain. There is "silver" level evidence that steroid injection may be more effective than ESWT [7,78]. Haake et al in a review of 20 studies concluded that no clinically relevant efficacy has been proven for the use of ESWT for lateral elbow pain [79,80]. Speed et al in a double blind randomized trial concluded that there appears to be a significant placebo effect of moderate dose of ESWT in subjects with lateral epicondylitis, but there is no evidence of added benefit of treatment when compared to sham therapy [82]. The differences were attributed to the patient selection, the techniques, the manufacture devices, the use of local anesthesia and the method of outcome measurements.

#### Calcifying tendinitis of the shoulder

The success rate of shockwave therapy in patients with calcific tendinitis of the shoulder was reported ranging from 78% to 91% [84-93]. Spindler et al reported complete pain relief and full shoulder joint movement in three patients two years after shockwave therapy, and a fragmentation of calcification was achieved after 24 h [12]. Wang et al compared the results of shockwave therapy in 37 patients (39 shoulders) with calcific tendonitis of the shoulder with a control group of 6 patients (6 shoulders). At 2- to 3-year follow-up, the overall results of the shockwave group were complaints free in 60.6%, significantly better in 30.3%, slightly better in 3.0% and unchanged in 6.1%. Only two patients (6%) showed recurrent pain of lesser intensity, and none showed worse symptoms. The results of the control group were slightly better in 1 (16.7%) and unchanged in 5 (83.3%). Radiographs showed complete elimination of calcium deposits in 57.6%, partial elimination or fragmentation in 15.1%, and unchanged in 27.3% for the shockwave group. For the control group, the calcium deposit was fragmented in 1 (16.7%) and unchanged in 5 (83.3%). None showed recurrence of calcium deposit 2 years after shockwave therapy. There was a correlation of functional improvement with the elimination of calcium deposit [94]. Jurgowski and Loew treated patients with two sessions of 2,000 impulses each of shockwave and reported a marked reduction of symptoms with an average of 30% improvement in the Constant score at the 12-week follow-up. Radiographs showed complete elimination of the calcification in seven patients, and partial elimination in five patients. Magnetic resonance imaging did not show any lasting damage to bone or soft tissue [95,96]. Rompe et al reported significant improvement in 72.5% of the patients and only six (15%) of 40 patients treated with 1,500 impulses of shockwaves reported no improvement. Complete or partial disintegration of the calcium deposits was observed in 62.5% of the patients [74]. In another study, Rompe et al reported that shockwave therapy provides equal or better results than surgery in patients with calcifying tendonitis of the shoulder [97].

#### Patellar tendinopathy (Jumper's knee) and Achilles tendinopathy

Several studies have reported favorable results of shockwave therapy in athletes with Jumper's knee (patellar tendinopathy) with the success rate ranged from 73.5% to 87.5% [16,19,43,98-100]. ESWT was also utilized in patients with patellar tendinopathy secondary to harvesting of the patellar tendon for ACL reconstruction. Wang et al compared 30 knees in 27 patients treated with ESWT with 24 knees in 23 patients treated conservatively, the results at 2- to 3-year follow-up showed 43% excellent, 47% good, 10% fair and none poor for the study group, and none excellent, 50% good, 25% fair and 25% poor for the control group (*P *< 0.05). Ultrasonographic examination showed a significant increase in the vascularity of the patellar tendon and a trend of reduction in the patellar tendon thickness after ESWT as compared to conservative treatments [43]. Peers KH et al compared 13 knees treated surgically with 15 knees received ESWT, and reported a comparable functional outcome in patient with patellar tendinopathy resistant to conservative treatments [100]. It appears that ESWT is effective in the management of patients with chronic patellar tendinopathy.

Many studies investigated the effect of ESWT in Achilles tendinopathy, and most reported favorable results with similar success rate as patellar tendinopathy [17,18,101-103]. Rompe et al compared 25 patients treated by eccentric stretching exercises with 25 patients treated with repetitive ESWT, and the results showed that eccentric loading is inferior to ESWT in the treatment of patients with chronic recalcitrant Achilles tendinopathy [101].

#### ESWT in bone disorders

##### Non-union and delayed union of long bone fracture

Several studies investigated the effect of shockwave therapy for non-union and delayed union of long bone fractures, and reported the success rate of achieving bony union ranged from 50% to 85% [13,14,104-110]. Schaden et al reported a success of 85% in the treatment of 115 delayed and non-unions [106]. Valchanou et al [107] reported bony unions in 70 of 82 patients with delayed or chronic nonunion of fractures at various locations. Vogel et al reported a 60.4% union rate in 48 patients with pseudarthroses treated with 3,000 shockwave impulses [108]. Wang et al treated 72 patients with non-unions of long bone fracture with shockwave therapy, and reported a success rate of 82.4% bony union at 6-month follow-up [104]. Rompe et al reported a 50% success rate in the treatment of delayed bone union with shockwaves in clinical study [109], whereas Schleberger and Senge [110] showed successful fracture healing in three of four pseudoarthroses treated with 2000 impulses of shockwaves. Recently, Elster EA et al reported an 80.2% success in 172 non-union of the tibia [14]. The results of ESWT in non-union of long bone appear to be comparable to surgical intervention. However, the advantages of ESWT include no surgery with no surgical pain and surgical risks.

##### AVNFH (Avascular necrosis of the femoral head)

For symptomatic hips affected by AVNFH, conservative treatments are generally not successful, and surgery is indicated with the type of surgery varying according to the stage of the disease [111]. Core decompression with or without bone grafting is considered the gold standard of femoral head preserving procedures. However, the results of core decompression varied widely and most reports are unsatisfactory [112] ESWT was recently utilized in the treatment of early AVNFH. Several articles reported the positive effect of shockwave therapy for AVNFH [21,22,113-116]. Wang et al compared 23 patients with 29 hips treated with ESWT and 25 patients with 28 hips treated by core decompression with non-vascularized fibular bone grafting, total hip arthroplasty (THA) was performed in 3% and 21% (*P *= 0.039) in 1 year, 10% and 32% (*P *= 0.044) in 2 years and 24% and 64% (*P *= 0.002) in 8 to 9-year follow-up for the ESWT group and the surgical group respectively. Significant improvements in pain and function were noted at each time intervals favoring the ESWT. There was a trend of decrease in the size of the lesion in the ESWT group [22,117]. In animal experiment in rabbits, ESWT was shown to increase BMP-2 protein and mRNA, and up-regulation of VEGF expression in necrotic subchondral bone of the femoral head. The up-regulation of VEGF may play a role inducing the ingrowth of neovascularization and improvement in blood supply to the femoral head [118,119]. These findings are in concert with our findings with histopathological examination and immunohistochemical analysis, ESWT was shown to promote angiogenesis and bone remodeling and regenerative effect in AVNFH [117]. It appears that ESWT is effective in the retardation or prevention of collapse of the femoral head in early AVNFH. The application of ESWT was also found effective in the treatment of corticosteriod induced AVNFH in patients with systemic lupus erythematosus [114]. Wang et al compared 15 patients with 26 hips in patients with systemic lupus erythematosus with the control of 24 patients with 29 hips, THA was performed in 12% and 14% respectively, and there were no difference in pain and function. It is concluded that the response of patients with SLE to ESWT for AVNFH is comparable to AVNFH in non-SLE patients [114].

##### Other disorders

Several studies reported a positive effect of shockwave therapy in Peyronie's disease and complex regional pain syndrome (RSD or reflex sympathetic dystrophy) [120], osteoarthritis of the knee [121], spine fusion [122], malignant cells [123,124], and gene therapy [125]. Furthermore, the application of ESWT has been expanded to non-musculoskeletal diseases. Recent studies showed that ESWT is effective in chronic diabetic foot ulcers [126,127] and ischemic heart disease [128,129].

In conclusion, ESWT is a new non-invasive therapeutic modality with effectiveness, convenience and safety. ESWT has the potential of replacing surgery in many orthopedic disorders without the surgical risks. The complication rates are low and negligible. The exact mechanism of shockwave therapy remains unknown. In animal experiments, ESWT induces a cascade of biological responses and molecular changes including the ingrowth of neovascularization and up-regulation of angiogenetic growth factors leading to the improvement in blood supply and tissue regeneration. There is a great potential for translational research and development in the armamentarium of extracorporeal shockwave technology.

## Competing interests

The author declared that he did not receive any honoraria or consultancy fee in writing this manuscript. No benefit was received or will be received directly or indirectly from a commercial party related to the performance of this study. The author has served as the member of scientific advisory committee of Sanuwave (Alpharetta, GA).

## Authors' contributions

C-JW participated in the study with the responsibility in protocol drafting, reference search, data collection and data analysis, manuscript writing and final proof of the manuscript.
